# Prognostic value of susceptibility-weighted imaging of prominent veins in acute ischemic stroke: A systematic review and meta-analysis

**DOI:** 10.3389/fneur.2022.1052035

**Published:** 2022-12-01

**Authors:** Wei Xiang, Zhigang Liang, Manman Zhang, Hongchun Wei, Zhongwen Sun, Yaodong Lv, Yuedan Meng, Wei Li, Huaguang Zheng, Hongxia Zhang

**Affiliations:** ^1^Department of Neurology, Yantai Yuhuangding Hospital Affiliated to Qingdao University, Yantai, China; ^2^The Second Clinical Medical College, Binzhou Medical University, Yantai, China; ^3^Department of Neurology, Beijing Tiantan Hospital, Capital Medical University, Beijing, China; ^4^China National Clinical Research Center for Neurological Diseases, Beijing, China

**Keywords:** acute ischemic stroke, meta-analysis, prognosis, prominent veins, susceptibility-weighted imaging (SWI)

## Abstract

**Background:**

The prominent veins sign (PVS) on susceptibility-weighted imaging (SWI) has been suggested to be related to the prognosis of patients with acute ischemic stroke (AIS). This meta-analysis aims to clarify the association between PVS and the prognosis of patients with AIS.

**Methods:**

This meta-analysis was registered in PROSPERO (no. CRD42022343795). We performed systematic research in PubMed, Web of Science, EMBASE, and Cochrane Library databases for studies investigating the prognostic value of PVS. Based on the enrolled studies, patients were divided into two groups as follows: those with PVS cohort and those without PVS cohort. Outcomes were unfavorable functional outcome, early neurological deterioration (END), and hemorrhagic transformation (HT). The random-effects models were used for the meta-analytical pooled. Heterogeneity was estimated using Cochran's *Q*-test and *I*^2^ value. Subgroup and sensitivity analyses were also performed to explore the potential sources of heterogeneity. Publication bias was assessed with funnel plots and using Begger's and Egger's tests.

**Results:**

A total of 19 studies with 1,867 patients were included. PVS was correlated with an unfavorable functional outcome in patients with AIS (risk ratio [RR] 1.61, 95% CI 1.28–2.02), especially in those receiving recanalization therapy (RR 2.00, 95% CI 1.52–2.63), but not in those treated conservatively (RR 1.33, 95% CI 0.87–2.04). Moreover, PVS was related to END (RR 2.77, 95% CI 2.21–3.46), while without an increased risk of HT (RR 0.97, 95% CI 0.64–1.47).

**Conclusion:**

PVS was associated with an unfavorable prognosis of patients with AIS and increased the risk of END, while not correlated with an increased risk of HT. PVS might be useful for predicting functional outcomes of patients with AIS as a novel imaging maker.

**Systematic review registration:**

https://www.crd.york.ac.uk/PROSPERO/, identifier: CRD42022343795.

## Introduction

Acute ischemic stroke (AIS) has been demonstrated to be the leading cause of morbidity and mortality worldwide, accounting for 87% of all strokes (1). It is also the most common cause of death in China due to the huge population, contributing to almost one-third of the total number of deaths due to stroke worldwide (2, 3). Early diagnosis, timely treatment, and improved prognosis in patients with ischemic stroke are vital requirements. Thus, identification of a prognostic imaging marker that could inform treatment decision-making and clinical outcome judging in the early stages of AIS has attracted great research interest.

Susceptibility-weighted imaging (SWI) is a blood oxygen level-dependent magnetic resonance technique developed as a useful tool for evaluating cerebral veins (4, 5). It is sensitive to paramagnetic substances and hence can indirectly reflect oxygen metabolism in tissues using the difference in the deoxygenated hemoglobin content between the hypoperfused region and normal tissues (6). Prominent veins sign (PVS) can be visualized as hypointensity and enlargement of vessels in the acute ischemic cerebral hemisphere with cortical and deep venous drainage (7). The prominence is thought to be an indicator of ischemic penumbra and is correlated with clinical prognosis. To date, 20 studies reported the relationship between PVS and the prognosis of patients with AIS, 15 of which believed that PVS had a worse prognosis than those without PVS (8–22), while the other 5 suggested that the presence of PVS was not related to prognosis (23–27). A previous meta-analysis has indicated that PVS was related to a poor functional prognosis in patients with AIS (28). However, it remains inconclusive whether PVS has an independent effect on prognosis in terms of pathophysiological factors such as large vessel occlusion. These studies generally have different study designs, patient characteristics, treatment administered to patients, small sample sizes, varying assessment of PVS, and heterogeneity of end points across studies. The purpose of this study was to provide a better understanding of the relationship between PVS and prognosis in patients with AIS.

## Materials and methods

### Search strategy

The study was performed according to the Preferred Reporting Items for Systematic Evaluation and Meta-Analysis guidelines and has been registered in PROSPERO (no. CRD42022343795). Our search strategy was based on a combination of the following keywords: “asymmetric^*^ or prominent or hypointense or cortical or medullary or deep cerebral” and “vessel^*^ or vein^*^” and “susceptibility-weighted or susceptibility weighted or SWI” and “stroke or cerebral infarction or cerebrovascular disease or brain infarction” (Supplementary Table 1). PubMed, Web of Science, Embase, and Cochrane Library were searched from inception to 1 April 2022. There was no language or other search restrictions. After identifying all potentially relevant studies, we removed duplicate studies using the Endnote X9 reference management software. Moreover, we reviewed the reference lists of the retrieved studies to ensure that no studies were omitted.

### Inclusion and exclusion criteria

The following eligibility criteria were used to reduce clinical heterogeneity: (1) patients aged ≥18 years and with an established diagnosis of AIS; (2) a baseline SWI was performed within 7 days after admission; (3) prognostic outcomes of patients with PVS and without PVS reported in eligible studies; and (4) the study design was a prospective or retrospective cohort study. We excluded (1) studies where outcomes were not separate for participants with and without PVS; (2) studies aim to investigate the relationship between PVS and other imaging features such as collateral circulation and perfusion status; and (3) all case series, case reports, letters, editorials, reviews, conference abstracts, guidelines, technical notes, and unrelated studies.

### Data extraction

Two authors (MZ and HW) independently reviewed the titles, abstracts, and full text of all the retrieved studies to determine their eligibility for inclusion. Disagreement was resolved by consensus with the corresponding author (ZL). If studies had overlapping samples, only the study with a larger sample size was included. Data extraction for each included study was done independently using a standardized electronic form. The discrepancies were resolved by consulting the third senior coauthor. The data extraction included (1) first author, publication year, study design, sample size, inclusion criteria, and clinical and imaging characteristics of enrolled patients at baseline (age, sex, hypertension, diabetes mellitus [DM], dyslipidemia, atrial fibrillation (AF), organic heart disease, history of stroke, time from stroke onset to SWI); (2) administration of research therapy (recanalization therapy, conservative treatment or mixed treatments); (3) PVS definition or assessment method: (i) visual assessment method, which classified patients with PVS if hypointense vessels were observed in the ipsilateral hemisphere compared with the contralateral side; (ii) patients scored quantitatively according to the Alberta Stroke Program Early CT Score and categorized into the PVS-positive group; (iii) slice assessment method (defining patients with ≥5 slice planes on 10 consecutive slices containing middle cerebral artery [MCA] regions as PVS-positive patients); and (iv) pixel comparison method (classifying PVS-positive patients by differences in venous signal intensity in bilateral cerebral hemispheres); and (5) primary and other outcomes in patients with and without PVS on baseline SWI. In the case of incomplete or unclear data, the authors were contacted using the details given in articles or identified by Internet search.

### Outcomes

The modified Rankin Scale (mRS) score will be used to assess the prognosis of patients with AIS. The primary outcome was an unfavorable functional outcome, defined as a score of 2–6 or 3–6 with no limit to the duration of follow-up. Other outcomes were 90-day unfavorable functional outcome, early neurological deterioration (END), and hemorrhagic transformation (HT). END was defined as neurological deterioration with an increase in the National Institutes of Health Stroke Scale (NIHSS) score ≥ 2 points in the first 72 h after admission. HT was determined by CT or MRI within 7 days after admission.

### Quality assessment

The Newcastle–Ottawa scale (NOS) was used to assess the quality of the included cohort studies, which included three factors, namely, selection, comparability, and exposure to assess the quality of each enrolled study. The NOS scores of 1–3, 4–6, and 7–9 indicate low, moderate, and high quality of studies, respectively. Two investigators (YM and HZ) independently conducted the quality assessment, and the differences were settled *via* consensus.

### Statistical analyses

All meta-analyses were performed using Stata software (v.16.0). A random-effects model was applied to calculate the pooled proportion meta-analysis. Pooled risk ratios (RRs) with 95% CI were estimated using the DerSimonian and Laird random-effects model. The data are presented as forest plots, with a two-sided *p*-value of <0.05 indicating statistical significance. We assessed statistical heterogeneity between studies using Cochran's *Q*-test (*p* < 0.1 indicating heterogeneity) and *I*^2^ statistic (*I*^2^ value of 25, 50, and 75% were considered as low, moderate, and high heterogeneity, respectively). The subgroup analysis of the primary outcome was based on the patients' treatment modalities, the types of PVS defined by location, and the stenosis rate of the intracranial artery. In addition, a random-effects meta-analysis was performed to show the association of clinical and imaging factors with the presence of PVS on baseline SWI. The publication bias was assessed using Begger's and Egger's tests of funnel plot symmetry in addition to visual inspection of the funnel plots.

## Results

### Literature research

**Figure 1:**
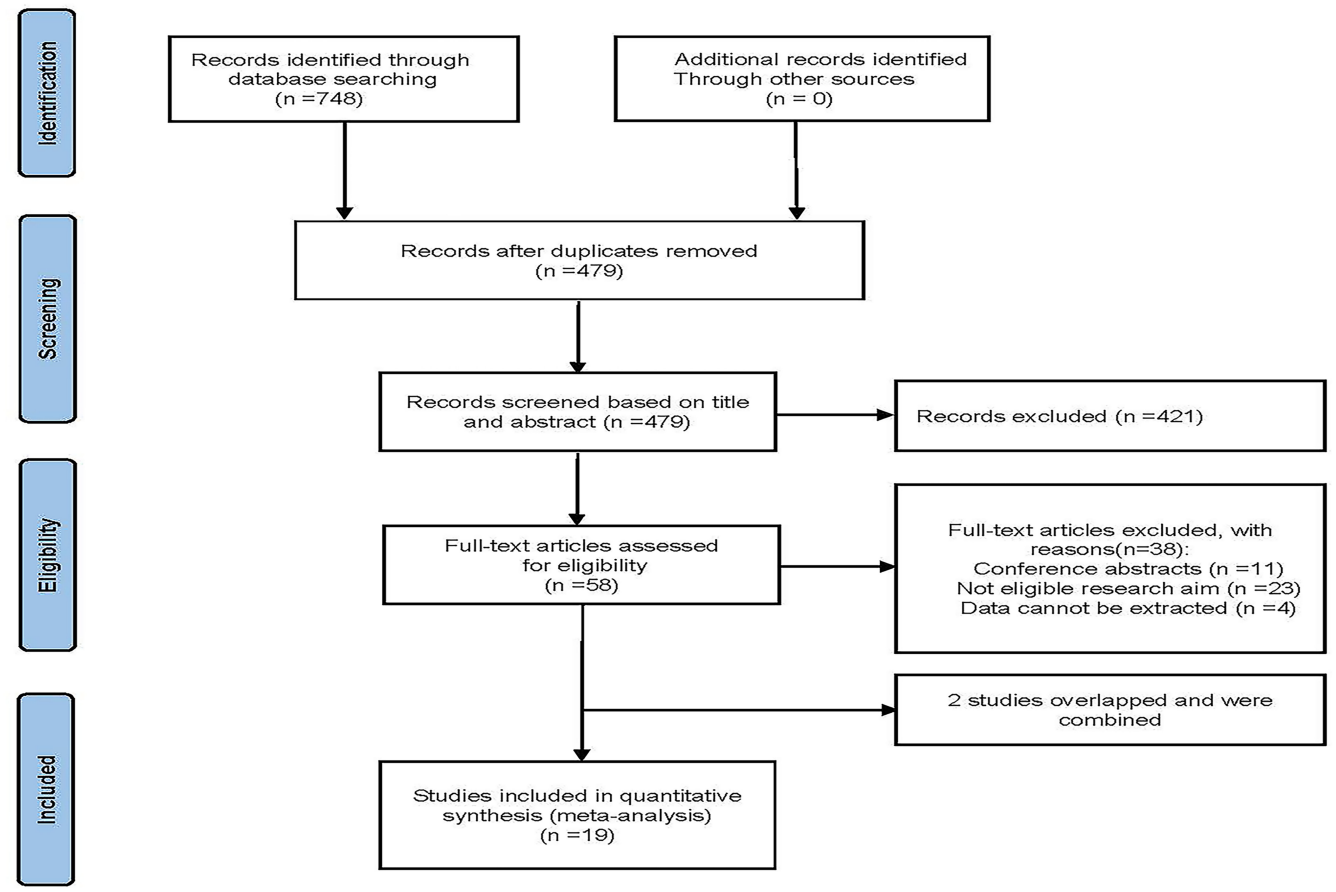
Flow diagram of the publication search and selection process.

Figure 1 shows the research flow diagram. The initial literature search provided a total of 748 relevant studies, of which 269 were excluded due to repetition. A total of 479 studies were reviewed by titles and abstracts, and 58 studies were included for full-text review. During the full-text assessment, 38 studies were excluded because they did not meet the eligibility criteria (*n* = 23), data could not be extracted from the study (*n* = 4), or studies were conference abstracts (*n* = 11). In the case of overlap in patient cohorts of the two studies, only the largest study was included in this systematic review (10, 11). Eventually, 19 cohort studies with 1,867 patients were included in our analysis (8–10, 12–27).

### Study characteristics

The included studies and relevant characteristics are provided in Table 1. The included studies were published between 2012 and 2022, and the sample sizes ranged from 22 to 572, with anterior circulation cerebral artery stenosis and infarction being the predominant type. A total of 7 studies were prospective in design, while 13 studies were retrospective. A total of 16 studies involving 1,488 patients (8, 10, 12–14, 16–21, 23–27) were pooled for an assessment of functional outcome, in which 13 studies involving 1,347 patients had this reported at 90 days (10, 12, 13, 16–21, 23, 25–27). Four studies (*n* = 324) (10, 12, 19, 20) were performed on recanalization therapy, three (10, 19, 20) of which were intravenous thrombolysis (IVT), and one (12) of which combined endovascular treatment (EVT) with IVT. Five studies (*n* = 951) reported END (9, 11, 13, 15, 22), while six studies (*n* = 352) reported the risk of HT (8, 10, 20, 24, 26). Data from 15 studies were pooled for influencing factors with the presence of PVS (8, 10, 12, 13, 15, 17–20, 22–27). The NOS scores ranged from 5 to 8, indicating a moderate-to-high quality of the included studies.

**Table 1:** Study characteristics and quality assessment of the included studies.

**Study**	**Study type**	**Sample size**	**Inclusion criteria**	**Time to SWI**	**Analysis method of PVS**	**Outcomes**	**Newcastle-Ottawa quality assessment scale**		
							**Selection**	**Comparability**	**Outcome**
Chen et al. (23)	P	22	MCA AIS patients	72h	ASPECT assessment	90-day mRs	⋆⋆⋆☆	☆☆	⋆⋆⋆
Huang et al. (24)	P	44	MCA AIS patients	48h	Visual assessment	180-day mRs, HT	⋆⋆⋆⋆	⋆☆	⋆⋆⋆
Jing et al. (8)	R	47	MCA AIS patients	8h	ASPECT assessment	7-day mRs, HT	⋆⋆⋆☆	☆☆	⋆⋆⋆
Li et al. (9)	R	109	AIS patients with SIASO	24h	Visual assessment	72-hour NI≥2	⋆⋆⋆☆	☆☆	⋆⋆☆
Liu et al. (25)	R	30	AIS patients with SIASO	72h	ASPECT assessment	90-day mRs	⋆⋆☆☆	⋆☆	⋆⋆⋆
Liu et al. (10)	P	55	AIS patients with SIASO	72h	Visual assessment	90-daymRs, HT	⋆⋆⋆☆	⋆⋆	⋆⋆⋆
Liu et al. (11)		61		72h	ASPECT assessment	48-hour NI≥2			
Oh and Lee (12)	R	100	AIS patients with SIASO	NR	ASPECT assessment	90-day mRs, HT	⋆⋆⋆☆	⋆⋆	⋆⋆⋆
Sun et al. (13)	P	572	MCA AIS patients	24h	Visual assessment	90-day MRS, 72-hour NI≥2	⋆⋆⋆⋆	⋆☆	⋆⋆⋆
Vural et al. (14)	R	50	AIS patients with MCA occlusion	24h	slice assessment	Discharge MRS	⋆⋆⋆☆	⋆☆	⋆⋆☆
Wang et al. (26)	R	46	AIS patients with MCA occlusion	48h	Visual assessment	90-daymRs, HT	⋆⋆☆☆	⋆⋆	⋆⋆⋆
Wang et al. (15)	R	76	AIS patients	12h	slice assessment	14-day NI≥2	⋆⋆⋆☆	⋆⋆	⋆⋆⋆
Wang et al. (16)	R	40	AIS patients with MCA occlusion	7d	Visual assessment	90-day mRs	⋆⋆⋆☆	⋆☆	⋆⋆⋆
Yu et al. (17)	R	124	AIS patients with MCA occlusion or stenosis	72h	Visual assessment	90-day mRs	⋆⋆☆☆	⋆☆	⋆⋆⋆
Yu et al. (18)	P	33	MCA AIS patients	7d	Pixel assessment	90-day mRs	⋆⋆⋆⋆	⋆☆	⋆⋆⋆
Zhang et al. (19)	R	109	AIS patients	4.5h	Pixel assessment	90-day mRs	⋆⋆⋆☆	⋆☆	⋆⋆⋆
Zhao et al. (20)	R	60	AIS patients	4.5h	Pixel assessment	90-day mRs, HT	⋆⋆☆	⋆☆	⋆⋆⋆
Hong et al. (21)	P	31	AIS patients with SIASO	24h	Visual assessment	90-day mRs	⋆⋆⋆⋆	⋆☆	⋆⋆⋆
Hu et al. (22)	R	133	AIS patients	72h	Visual assessment	7-day NI≥2	⋆⋆⋆☆	☆☆	⋆⋆☆
Jia et al. (27)	P	125	MCA AIS patients	7d	Visual assessment	90-day mRs	⋆⋆⋆⋆	⋆☆	⋆⋆⋆

The “Liu et al. (10, 11)” cohort populations overlapped, and the two studies were combined.

P, prospectively; R, retrospective; NR, not reported; PVS, prominent vessel sign; SWI, susceptibility-weighted imaging; AIS, acute ischemic stroke; MCA, middle cerebral artery; SILASO, severe intracranial large artery stenosis or occlusion; NI, NIHSS, National Institutes of Health Stroke Scale; NI≥2, an increase in total NIHSS score of ≥2, early neurological deterioration; mRS, modified Rankin scale; HT, hemorrhagic transformation; ASPECT, Alberta Stroke Program Early CT score.

⋆ Score 1 of the Newcastle-Ottawa quality assessment scale. ☆ Score 0 of the Newcastle-Ottawa quality assessment scale.

### Association of baseline factors with the presence of PVS

The factors related to the presence of PVS are shown in Table 2. Prior stroke/transient ischemic attack (TIA) (RR 1.34, 95% CI 1.02–1.75, *p* = 0.034) and AF (RR 1.43, 95% CI 1.04–1.97, *p* = 0.026) had an independent relationship with the presence of PVS. Furthermore, severe intracranial large-artery stenosis/occlusion (SILASO) was significantly associated with the presence of PVS (RR 1.84, 95% CI 1.37–2.47, *p* < 0.001).

**Table 2:** Meta-analysis of the associations of baseline factors with PVS.

**Factors**	**Studies (Patients)**	**Reference number of included studies**	**Risk ratio or mean difference (95% CI)**	**Heterogeneity I2 (%)**
Age (year) (with PVS-SWI vs. without)	14 (1443)	(8, 10, 12, 13, 15, 17–20, 23–27)	−0.34(−2.41,1.73)	38
Male vs. female	15 (1,576)	(8, 10, 12, 13, 15, 17–20, 22–27)	1.04 (0.95,1.13)	0
With smoking vs. without	9 (1,263)	(10, 12, 13, 15, 18–20, 22, 27)	0.97 (0.78,1.22)	21
With prior stroke/TIA vs. without	7 (658)	(10, 12, 15, 19, 20, 22, 27)	1.34 (1.02,1.75)	0
With hypertension vs. without	12 (1,477)	(10, 12, 13, 15, 17–20, 22, 24, 26, 27)	0.97 (0.86,1.10)	44
With DM vs. without	12 (1,477)	(10, 12, 13, 15, 17–20, 22, 24, 26, 27)	0.93 (0.77,1.12)	0
With dyslipidemia vs. without	8(1,193)	(12, 13, 17, 18, 20, 22, 26)	1.09 (0.90,1.31)	19
With AF vs. without	10 (1,400)	(10, 12, 13, 15, 17, 19, 20, 22, 26, 27)	1.43 (1.04,1.97)	34
With organic heart disease vs. without	4 (890)	(13, 20, 22, 27)	0.93 (0.61,1.42)	0
With SILASO vs. without	8 (1,219)	(8, 13, 15, 17–19, 22, 27)	1.84 (1.37,2.47)	84

PVS, prominent vessel sign; TIA, transient ischemic attack; AF, atrial fibrillation; DM, diabetes mellitus; SILASO, severe intracranial large artery stenosis or occlusion.

### Association of PVS with functional outcome

**Figure 2:**
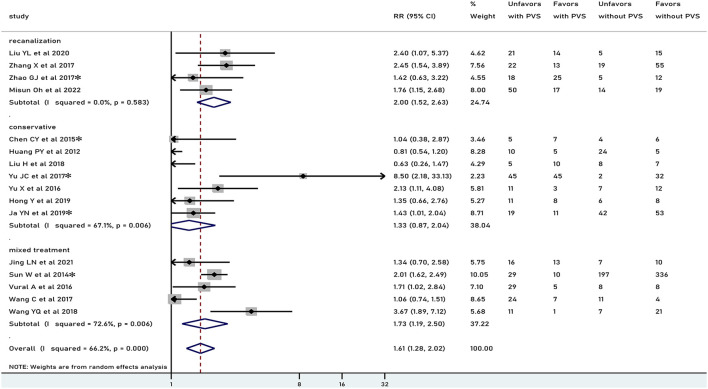
Meta-analysis of associations between PVS and unfavorable functional outcome by modality of treatment to the patients. PVS, prominent vessel sign; Unfavors, unfavorable functional outcome; Favors, favorable functional outcome; *mRS scores 2–6 were regarded as unfavorable outcome (mRS scores 3–6 for other studies).

**Figure 3:**
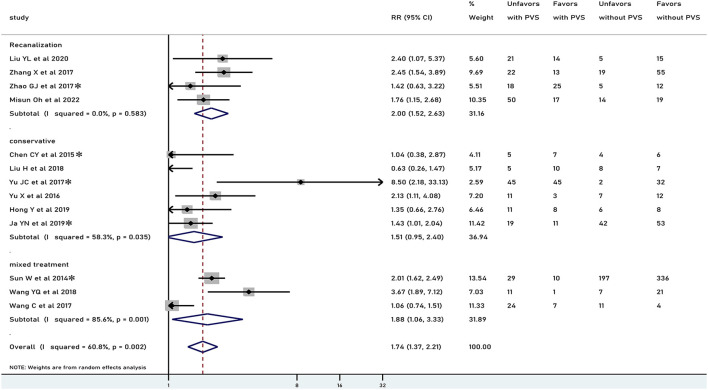
Meta-analysis of associations between PVS and 90-day functional outcome by modality of treatment to the patients. PVS, prominent vessel sign; Unfavors, unfavorable functional outcome; Favors, favorable functional outcome; *mRS scores 2–6 were regarded as unfavorable outcome (mRS scores 3–6 for other studies).

**Figure 4:**
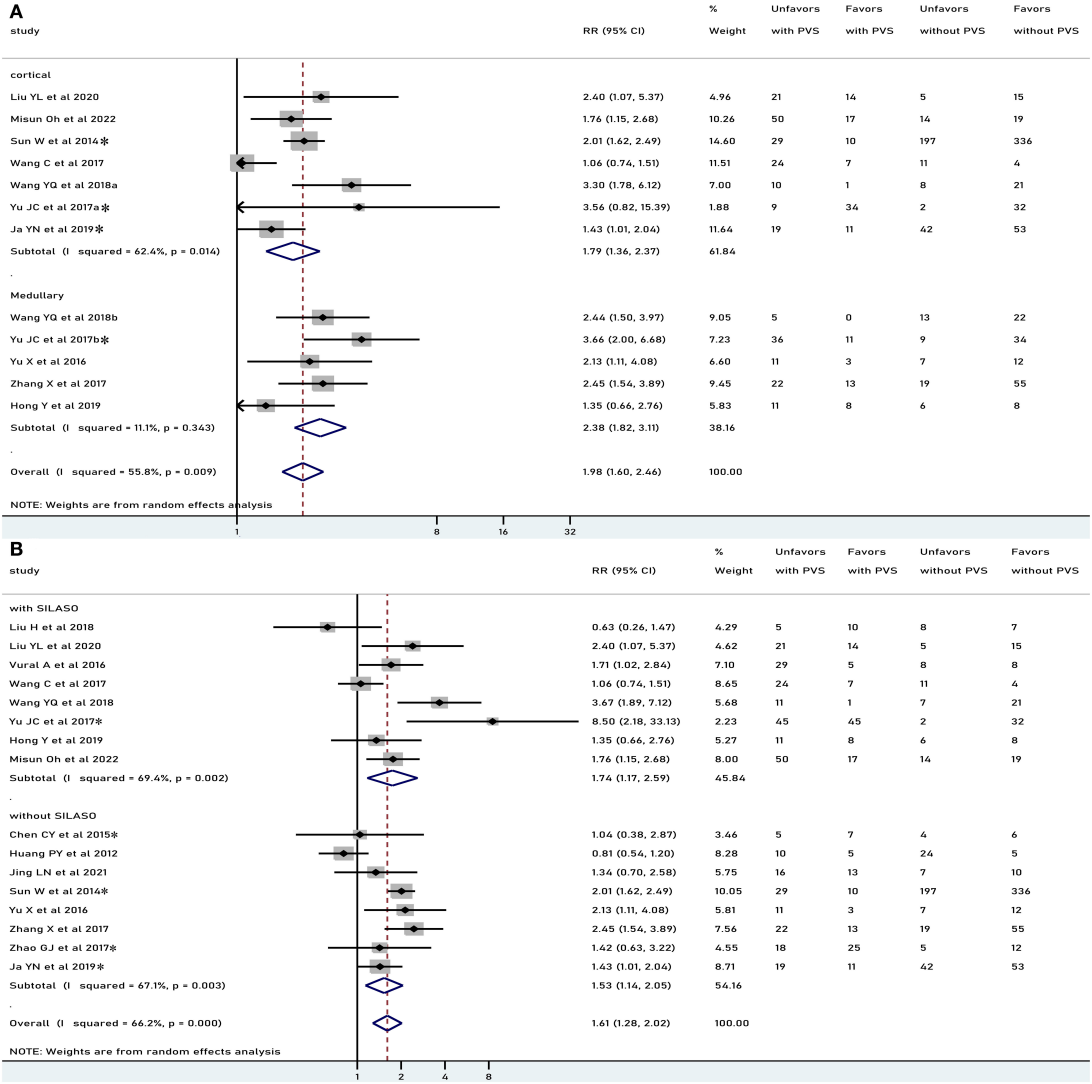
Meta-analysis of associations between location of PVS and unfavorable functional outcome **(A)** and meta-analysis of association between PVS and unfavorable functional outcome, by different stenosis of intracranial artery to the study patients **(B)**. PVS, prominent vessel sign; Unfavors, unfavorable functional outcome; Favors, favorable functional outcome; *mRS scores 2–6 were regarded as unfavorable outcome (mRS scores 3–6 for other studies).

In general, PVS was associated with an unfavorable functional outcome (RR 1.61, 95% CI 1.28–2.02, *p* < 0.001), especially in patients who underwent recanalization treatment (RR 2.00, 95% CI 1.52–2.63, *p* < 0.001), but not in patients treated conservatively (RR 1.33, 95% CI 0.87–2.04, *p* = 0.186) (Figure 2). There was moderate heterogeneity in results pooled for functional outcome (*I*^2^ = 66.2%). Comparable results were obtained in a pooled analysis of the 90-day functional outcome (Figure 3). The subgroup was stratified by the location of the PVS. As shown in Figure 4A, we found that the differences in the cortex or medulla were related to a poorer functional prognosis (RR 1.79, 95% CI 1.36–2.37, *p*<0.001, *I*^2^ = 62.4%/RR 2.38, 95% CI 1.82–3.11, *p* < 0.001, *I*^2^ = 11.1%). There were similar associations in subgroup analyses by stenosis rate of the intracranial artery and functional outcome: being associated with an unfavorable outcome for patients with SILASO (RR 1.74, 95% CI 1.17–2.59, *p* =0.006) and without SILASO (RR 1.53, 95% CI 1.14–2.05, *p* =0.005) (Figure 4B). Heterogeneity for associations was generally moderate across studies (*I*^2^ = 69.4/*I*^2^ = 67.1).

### Association of PVS with other outcomes

**Figure 5:**
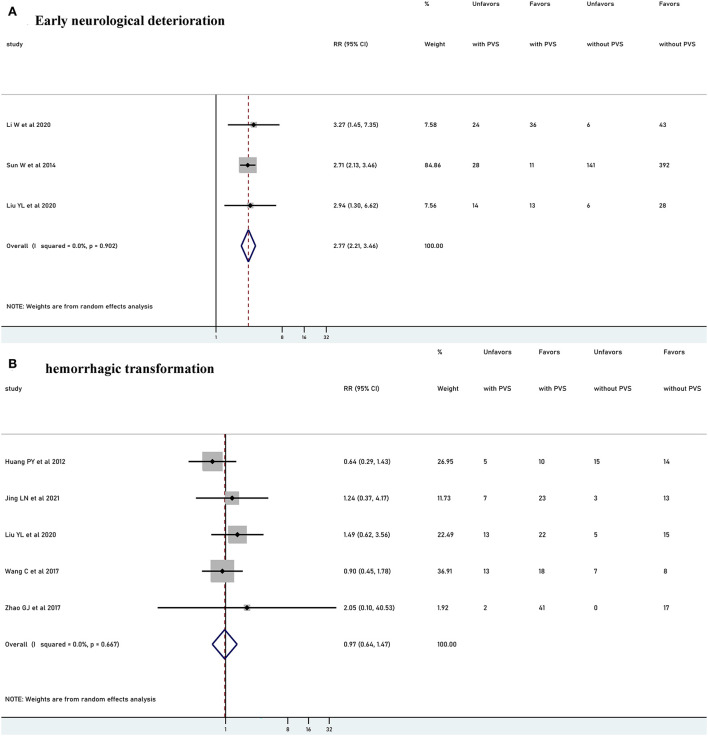
Meta-analysis of relationships between PVS and early neurological deterioration **(A)** and meta-analysis of relationships between PVS and hemorrhagic transformation **(B)**. PVS, prominent vessel sign; Unfavors, unfavorable functional outcome; Favors, favorable functional outcome.

Five studies with 951 patients reported the relationship between PVS and END. Two studies were excluded from the meta-analysis due to the different criteria time of END. PVS was significantly associated with increased risk of END (RR 2.77, 95% CI 2.21–3.46, *p*<0.001, *I*^2^ = 0%) (Figure 5A). Six studies (*n* = 352) explored the relationship between PVS and HT. Among them, one study was excluded from the meta-analysis due to the definition time of HT was unclear. There was no significant association with HT (RR 0.97, 95% CI 0.64–1.47, *p* = 0.889, *I*^2^ = 0%) (Figure 5B).

### Publication bias

The funnel plot was symmetrical, indicating no publication bias in this meta-analysis. The Begg's and Egger's tests were insignificant, indicating no publication bias (Supplementary Figures 1–4).

## Discussion

In this systematic review and meta-analysis, we have identified the key factors determining the presence of PVS that were previous stroke/TIA, AF, and SILASO. While overall PVS was associated with an unfavorable outcome especially when recanalization treatment was performed, but not in patients treated conservatively. PVS was also associated with END but without an increased risk of HT. Those results may be helpful for clinicians to identify the high-risk populations with poor prognosis after AIS, thus providing timely and appropriate interventions.

The incidence of PVS has been reported to be ranging from 6.8 to 64% (16, 23, 29). Patients with cerebral artery stenosis or occlusion were more likely to present with PVS (30–32). Our results suggest that PVS was significantly related to SILASO, which was consistent with the published findings. The underlying mechanism of PVS was an imbalance that occurred between cerebral oxygen supply and demand in the infarct area after AIS, which caused an increased level of deoxyhemoglobin in the vessels (33). Severe stenosis/occlusion of the cerebral arteries led to a more significant decrease in cerebral blood flow to the distal tissues, resulting in a more pronounced increase in deoxyhemoglobin. Furthermore, our analysis showed that the presence of PVS was also associated with a history of AF. One explanation may be that embolization could cause stenosis of large arteries in patients with AF (34). Association between PVS and previous stroke/TIA may be supported by the fact that patients with a previous stroke were more likely to have stenosis in the large cerebral arteries.

Our research demonstrated that PVS was significantly associated with poor prognosis in those who received recanalization therapy. Early recanalization has been proven to be the first line of therapy to improve the outcome of patients with AIS (35). However, there was a significant number of patients with poor outcomes despite successful recanalization (36, 37). A growing body of evidence suggests that the perfusion status of ischemic brain regions was closely related to the prognosis after recanalization (38, 39). To some extent, PVS indicated a state of perfusion in AIS that reduces the efficacy of recanalization and increases the risk of poor prognosis (20). Bilk et al. (40) reported that all 10 patients with hyperacute ischemic stroke complicated by large-artery occlusion showed notable PVS before recanalization therapy, while there were no longer observed in SWI after recanalization. PVS can be reversed or disappear by timely reperfusion, and hence can be used as an indicator to evaluate the effect of IVT or EVT to guide individualized treatment (41). Therefore, PVS still existing after treatment may be a sign of poor patient prognosis (42, 43). Notably, it is not recanalization therapy itself that leads to poor prognosis as our study showed that PVS did not increase the risk of HT. Conversely, recanalization led to more obvious differences in poor outcomes between PVS-positive and PVS-negative patients. Thus, recanalization should not be delayed owing to the appearance of PVS. Given the narrow time window constraints (<4.5 h for IVT or <6 h for EVT), the time of SWI examination was not uniform in the included studies, which limited the robustness of these results. Future stratified research is warranted with larger sample sizes.

Interestingly, the pooled results for PVS and poor prognosis in the conservative treatment group were not significant. On the one hand, it was believed that if the hypoperfusion state was not improved in time in patients with PVS, the structural integrity of brain cells was irreversibly damaged with the prolongation of the disease. Eventually, the cells lost their oxygen uptake capacity and died. At the same time, vasodilatory regulation gradually weakened, and the venous structures collapsed (44). Both these changes were manifested as the disappearance of PVS on SWI, decreasing the difference in the poor prognosis between patients with and without PVS. In contrast, it appeared that patients were treated conservatively because they were beyond the time window for recanalization and had a higher incidence of contraindications. Compared with the recanalization group, the aforementioned populations had more underlying diseases and a worse overall prognosis.

The subgroup analysis based on location revealed that both cortical and medullary veins were related to an unfavorable outcome. Medullary veins are better than cortical veins as predictors of poor prognosis. A possible pathophysiological factor may be that compared with cortical PVS, the presence of medullary PVS reflects the increase in the deoxyhemoglobin level in the draining deep medullary veins above a limit of detection, suggesting the decompensation of oxygen capacity, which is more likely to be reached in severe strokes (45, 46). Subsequent studies indicated that compared with patients with cortical PVS alone patients with both cortical and medullary PVS had larger infarct sizes and more severe neurological impairments, which was also consistent with our findings (8). Medullary PVS may be more sensitive in predicting early poor prognosis. Hence, successful reperfusion therapy may be more urgent for patients with medullary PVS. Many studies have shown that PVS occurred more in patients with SILASO, which suggests that SILASO should be taken into account in the analysis of patients with AIS. In the subgroup analysis of stenosis rate, we found that the PVS is associated with a poor functional prognosis regardless of whether the patient combined with SILASO. Moreover, patients with SILASO may be at higher risk of a poor outcome compared with without SILASO, which is consistent with prior studies reporting patients with AIS due to intracranial atherosclerosis stenosis or occlusion have a higher risk of unfavorable outcome (47).

This study had several limitations. First, the included studies were observational studies, leading to a selection bias. Second, due to the low number of studies and sample size related to END and HT, further meta-analyses with larger samples are needed.

## Conclusion

Our study suggests that PVS was closely related to the poor prognosis of patients with AIS treated with recanalization. Among the PVS-positive population, the efficacy of aggressive reperfusion therapy was not significant. However, we believe that reperfusion therapy should not be postponed because of the presence of PVS. PVS is valuable as a noninvasive imaging marker for predicting prognosis and warrants further investigation.

## Data availability statement

The original contributions presented in the study are included in the article/Supplementary material, further inquiries can be directed to the corresponding author/s.

## Author contributions

ZL and WX conceived the idea and designed the research. MZ and HW searched databases and performed initial screening. YM and HZha performed data extraction and interpretation. ZS and YL performed the statistical analysis. WX wrote the first draft of the manuscript. ZL, WL, and HZhe revised the manuscript. All the authors read and approved the final version of the manuscript for publication.

## Conflict of interest

The authors declare that the research was conducted in the absence of any commercial or financial relationships that could be construed as a potential conflict of interest.

## Publisher's note

All claims expressed in this article are solely those of the authors and do not necessarily represent those of their affiliated organizations, or those of the publisher, the editors and the reviewers. Any product that may be evaluated in this article, or claim that may be made by its manufacturer, is not guaranteed or endorsed by the publisher.

## Abbreviations

PVS: Prominent veins sign
SWI: Susceptibility-weighted imaging
AIS: Acute ischemic stroke
END: Early neurological deterioration
HT: Hemorrhagic transformation
IVT: Intravenous thrombolysis
EVT: Endovascular thrombectomy.
