# Supplementary material for: Prognostic value of susceptibility-weighted imaging of prominent veins in acute ischemic stroke: A systematic review and meta-analysis
Source: Front Neurol. 2022 Dec 1;13:1052035. doi: 10.3389/fneur.2022.1052035 (PMC9751901; doi:10.3389/fneur.2022.1052035)

Supplementary Material

# Supplementary Tables

**Supplementary Table S1.** Search strategy of present systematic review and meta-analysis.

| PubMed | ((hypointense) OR (prominent) OR (asymmetric*) OR (cortical) OR (medullary) OR (deep cerebral)) AND ((vessel*) OR (vein*)) AND ((SWI) OR (susceptibility-weighted) OR (susceptibility weighted)) AND ((stroke) OR (cerebral infarction) OR (cerebrovascular disease) OR (brain infarction)) |
| --- | --- |
| Web of science | ALL=(“hypointense” OR “prominent” OR “asymmetric*” OR “cortical” OR “medullary” OR “deep cerebral”) AND ALL=(“vessel*” OR “vein*”) AND ALL=(“SWI” OR “susceptibility-weighted” OR “susceptibility weighted”) AND ALL=(“stroke” OR “cerebral infarction” OR “cerebrovascular disease” OR “brain infarction”) |
| Embase (Ovid SP): | (“hypointense” OR “prominent” OR “asymmetric*” OR “cortical” OR “medullary” OR “deep cerebral”) AND (“vessel*” OR “vein*”) AND (“SWI” OR “susceptibility-weighted” OR “susceptibility weighted”) AND (“stroke” OR “cerebral infarction” OR “cerebrovascular disease” OR “brain infarction”) |
| Chochrane: | (ALL (“hypointense”) OR ALL (“prominent”) OR ALL(“asymmetric*”) OR ALL(“cortical”) OR ALL(“medullary”) OR ALL(“deep cerebral”)) AND (ALL (“vessel*”) OR ALL (“vein*”)) AND (ALL (“SWI”) OR ALL (“susceptibility-weighted”) OR ALL (“susceptibility weighted”)) AND (ALL (“stroke”) OR ALL (“cerebral infarction”) OR ALL (“cerebrovascular disease”) OR ALL (“brain infarction”)) |
| Chinese Biomedical Literature Database | ((hypointense) OR (prominent) OR (asymmetric*) OR (cortical) OR (medullary) OR (deep cerebral)) AND ((vessel*) OR (vein*)) AND ((SWI) OR (susceptibility-weighted) OR (susceptibility weighted)) AND ((stroke) OR (cerebral infarction) OR (cerebrovascular disease) OR (brain infarction)) |

# Supplementary Figures

**Supplementary Figure S1.** Funnel plot of included studies for any unfavorable functional outcome between with PVS group or without PVS group. Begg’s test (*p*=0.822) and Egger’s test (*p*=0.955), which are insignificant indicating no publication bias.


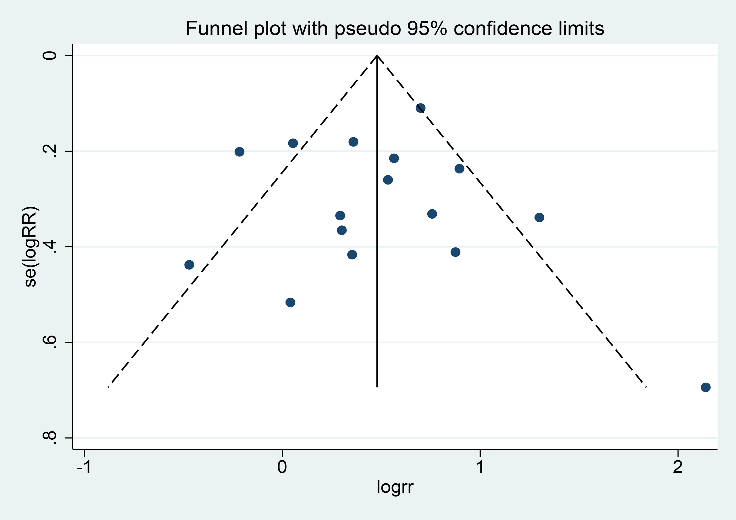


**Supplementary Figure S2.** Funnel plot of included studies for 90-days unfavorable functional outcome between with PVS group or without PVS group. Begg’s test (*p*=0.837) and Egger’s test (*p*=0.936), which are insignificant indicating no publication bias.


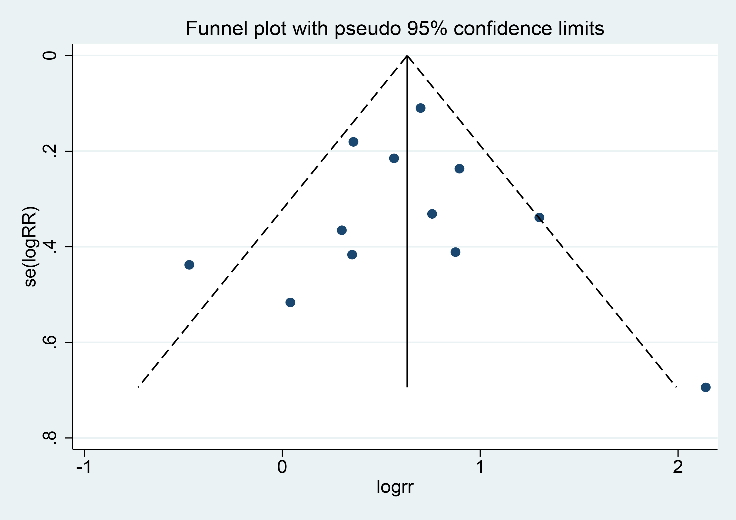


**Supplementary Figure S3.** Funnel plot of included studies for early neurological deterioration between with PVS group or without PVS group. Begg’s test (*p*=1.000) and Egger’s test (*p*=0.261), which are insignificant indicating no publication bias.


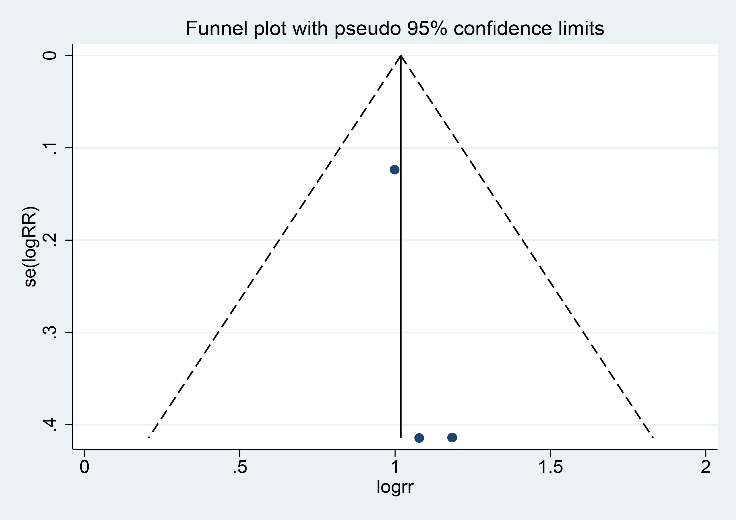


**Supplementary Figure S4.** Funnel plot of included studies for hemorrhagic transformation between with PVS group or without PVS group. Begg’s test (*p*=0.462) and Egger’s test (*p*=0.402), which are insignificant indicating no publication bias.


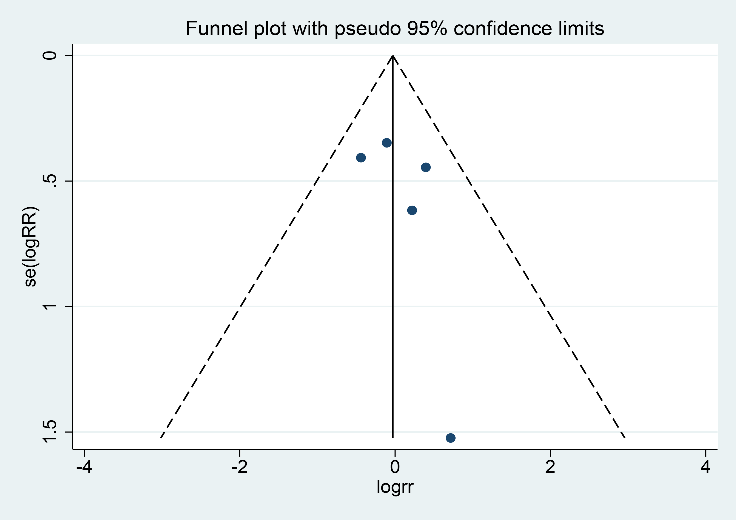

Supplement: Supplementary file 1 [file Data_Sheet_1.docx]
